# Size Matters: A Latent Class Analysis of Workplace Health Promotion Knowledge, Attitudes, Practices and Likelihood of Action in Small Workplaces

**DOI:** 10.3390/ijerph17041251

**Published:** 2020-02-15

**Authors:** Geneviève Jessiman-Perreault, Amanda Alberga, Fatima Jorge, Edward Makwarimba, Lisa Allen Scott

**Affiliations:** 1 Alberta Cancer Prevention Legacy Fund; Alberta Health Services, Calgary, AB T2S 3C3, Canada; amanda.alberga@albertahealthservices.ca (A.A.); fatima.jorge@albertahealthservices.ca (F.J.); lisa.allenscott@albertahealthservices.ca (L.A.S.); 2 Faculty of Medicine and Dentistry, University of Alberta, Edmonton, AB T6G 2R3, Canada; em@ualberta.ca

**Keywords:** workplace health, small workplace, health promotion

## Abstract

Workplace health programs (WHPs) have been shown to improve employee health behaviours and outcomes, increase productivity, and decrease work-related costs over time. Nonetheless, organizational characteristics, including size, prevent certain workplaces from implementing these programs. Past research has examined the differences between small and large organizations. However, these studies have typically used a cut-off better suited to large countries such as the USA. Generalizing such studies to countries that differ based on population size, scale of economies, and health systems is problematic. We investigated differences in WHP knowledge, attitudes, and practices between organizations with under 20 employees, 20–99 employees, and more than 100 employees. In 2017–2018, a random sample of employers from 528 workplaces in Alberta, Canada, were contacted for participation in a cross-sectional survey. Latent Class Analysis (LCA) was used to identify underlying response pattern and to group clusters of similar responses to categorical variables focused on WHP knowledge, attitudes, practices and likelihood of action. Compared to large organizations, organizations with fewer than 20 employees were more likely to be members of the Medium–Low Knowledge of WHP latent class (*p* = 0.01), the Low Practices for WHP latent class (*p* < 0.001), and more likely to be members of Low Likelihood of Action in place latent class (*p* = 0.033). While the majority of workplaces, regardless of size, recognized the importance and benefits of workplace health, capacity challenges limited small employers’ ability to plan and implement WHP programs. The differences in capacity to implement WHP in small organizations are masked in the absence of a meaningful cut-off that reflects the legal and demographic reality of the region of study.

## 1. Introduction

Workplaces are an important setting for health promotion [1,2,3]. Approximately 66% of adult Canadians are in the workforce and the typical worker will spend more time at work compared with any other setting [4]. Leveraging the workplace as a priority setting for health promotion presents a unique opportunity to engage a diverse population, with the further potential to reach subsets of the population that are difficult to engage through traditional means [5,6]. Workplace environments can be health promoting or constraining as they directly influence the physical, mental, economic, and social well-being of individual employees and, as a result, the health of their families, communities, and society [5]. Adverse working conditions can lead to physical and psychosocial health hazards, and this relationship has a gradient effect based on socioeconomic status, where lower-status occupations are associated with higher risk for accidents and illnesses [5]. Therefore, working conditions contribute significantly to health inequities [7]. Poor working conditions include health exposures in the physical environment such as injuries and occupational diseases; psychosocial conditions that foster stress and poor mental health such as pace, demands, or repetitive nature of the job; stressful conditions that impact employees’ lives outside of work and their connection with the community [8]; unsupportive work environments that do not provide employees with the resources and supports to reduce modifiable risk factors for chronic disease [6].

Typically, workplaces adopt workplace health programs, defined as the collaborative efforts of employers, employees, communities and society to improve the health and well-being of people at work [6]. These programs comprise of physical and psychological well-being, coupled with existing safety programs and policies, to create a health promoting environment. Adopting these types of programs has been shown to improve employee health behaviours and outcomes, increase job satisfaction morale and productivity, decrease absenteeism, presenteeism and other work-related costs over time [3,9,10]. Despite the potential value of such programs, organizational characteristics prevent certain workplaces from implementing these programs.

Numerous research studies have concluded that small to midsized businesses (defined anywhere between 100 and 5000 employees) are significantly less likely to offer health promotion programs [1,11,12,13,14]. Perceived barriers to implementation among small businesses include direct cost of implementation [13], indirect costs such as time and staffing [6], lack of employee interest [9], lack of management support [13], lack of knowledge, skill or expertise [1,3,12], and difficulties with evaluation [12]. Moreover, smaller workplaces may feel overburdened by occupational health and safety regulations and may avoid implementing health promotion programs if they are not mandated [15]. While smaller workplaces report numerous barriers in implementing a workplace health program (WHP), they may also have characteristics that promote implementation such as a less bureaucratic structure, fewer employees creating more opportunities to accommodate preferences, more personal accountability, potential for teamwork and group bonding through the development and implementation period [12]. Interestingly, past research on workplace health promotion found that employee participation rates among small businesses were higher due to incentivization [1,3,6,16,17].

While an abundance of research exists on the difference in perceived ability to implement workplace health promotion programs between small and large workplaces [12], these studies typically occur in the USA using a cut-off for small business of anywhere from 100 to 5000 [13,15,16,18]. Generalizing these results to a Canadian context is inappropriate for two reasons: first, differences in the structure and delivery of health care between Canada and the USA has meaningful impacts on employers’ willingness to invest in WHP. Most of the USA’s population is covered by private health insurance purchased by individuals or employers therefore employers may be more willing to invest in WHP to reduce their insurance premiums [19]. In countries with universal health coverage, such as the United Kingdom, Canada and Australia, interest in implementing WHP is lower [3]. Second, the size of organizations, and populations, in Canada are much smaller than the USA. Innovation, Science and Economic Development Canada (ISED) defines a small business as having under 100 employees [20], whereas the Office of Advocacy defines a small business as less than 500 employees [21]. In contrast, nations with population distribution and health care delivery systems similar to Canada, such as Australia and New Zealand, employ more comparable cut-offs of under 15 [22] and under 20 [16,23], respectively. Recently, lower-range classifications of small workplaces (10–24, 25–49, 50–99) have been incorporated in a large survey conducted in the United States of America [1,14].

While the Canadian threshold for small businesses (< 100 employees) is much smaller than the USA, it may still be inaccurate for assessing barriers in implementation of WHP and could be masking the depth of inequities in ability to implement WHP between workplaces of varying sizes. In Alberta, 88% of businesses have under 20 employees; and in Canada, 87% of business have under 20 employees [24]. Within Canada, following in the footsteps of Ontario which implemented similar legislation, Alberta amended their Occupational Health and Safety Act to include requirements that all workplaces with more than 20 employees have a Joint Workplace Health and Safety Committee and a policy protecting employees from bullying and harassment [25]. These changes were an important step in mandating workplaces to implement an integrated approach to employee health and safety. Given the focus on workplace size, it is important to assess workplaces’ ability to implement these changes to inform the development of a support model to assist them in implementing WHP.

Within this paper we examine current knowledge, attitudes, and practices of Alberta employers regarding workplace health, and determine whether differences exist between the knowledge, attitudes, practices, and likelihood of action of employers to implement WHP in their organization by organization size. This study is important because it provides new insights into the differences in WHP related knowledge, attitudes and practices of Alberta workplaces using three cut-offs for organization size (<20 employees, 20–99 employees, and more than 100 employees). By using an organizational size cut-off that reflects the demographic, economic, and legislative realities of the geographic area understudy, this study provides a new understanding of the WHP knowledge, attitudes, practices, and likelihood of action of small organizations that might be otherwise masked by using an inaccurate size threshold.

## 2. Materials and Methods

### 2.1. Survey Instrument

**Figure 1:**
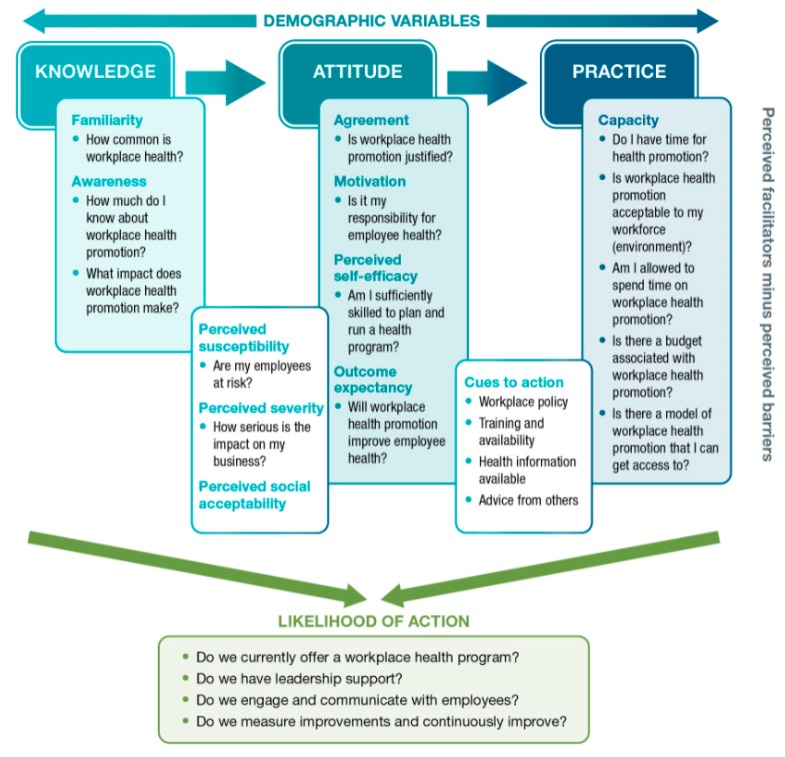
Conceptual Framework of Knowledge, Attitude and Practices as Determinants for Workplace Health Promotion Likelihood of Action. Adapted from [26].

We developed a novel survey instrument informed by the knowledge, attitudes, and practices (KAP) conceptual framework, presented in Figure 1 [26,27]. This KAP framework posits that a combination of key constructs—organizational knowledge, attitudes, and practice—leads to likelihood of action. In this framework, knowledge is defined as the factual information from training and experience. Knowledge includes the concepts of familiarity and awareness. In our instrument, knowledge is measured through true and false response to a variety of statements on WHP in general. Attitudes are defined as an affect (an emotion, feeling or desire) and include the concepts of perceived susceptibility, perceived severity, perceived social acceptability, agreement, motivation, perceived self-efficacy and outcome expectancy. Attitudes are measured through a 4-point Likert scale which assesses agreement with a variety of statements about the employers’ perception of WHP in their own organization. Practices are defined as the perceived capacity to take action to achieve a certain workplace health goal. Practices include perceived barriers and enablers including human resources, time, funding and previous experience in the organization and is measured using a 4-point frequency scale (often, sometimes, rarely, never). Taken together these constructs lead to the likelihood of action [3,28] defined as currently offering a WHP program as well as the approach taken to implementation and is measured using a 4-point frequency scale (often, sometimes, rarely, never).

In alignment with this conceptual framework, survey questions were developed based on a 2-pronged literature review. The first literature focused on identifying previously evaluated workplace health surveys. We were unable to find a specific and validated survey that measured knowledge, attitudes and practices for WHP. Therefore, the second literature review focused on examining the components on the KAP conceptual framework (Figure 1) to better understand the barrier and enablers to implementing WHP [3,28,29,30,31,32,33]. For a more detailed explanation of the questions included in the survey instrument, the scale of measure, and the concepts included, see Appendix A.

Construct reliability of the survey instrument was assessed using Cronbach’s alpha and composite reliability to determine internal consistency, the extent to which all items in a questionnaire contribute towards measuring the same construct. Cronbach’s alpha was calculated for each key construct (i.e., knowledge, attitudes, practices, and likelihood of action) separately. Given the exploratory nature of this research, we used a Cronbach’s alpha cut-off of ≥0.60 as proposed by Hair and associates [34]. The items assessing knowledge, attitudes, practices, and likelihood of action yielded satisfactory Cronbach’s alphas of 0.63, 0.78, 0.82, and 0.88, respectively.

Factor analysis, using varimax rotation, was conducted on each key construct using a rotated factor loading of ≥0.40 as a cut-off [35]. There were 2 factors loaded for knowledge, 2 factors loaded for attitudes, 1 factor loaded for practices and 1 factor loaded for likelihood of action. Two items, one attitudes item and one practices item, did not load on any factors and were removed from the survey instrument. Convergent validity of the survey instrument was assessed by calculating the average variance extracted (AVE) and composite reliability using standard loadings from the factor analysis. Typically, acceptable AVE is defined as scores ≥0.50. Using this definition, both factors for knowledge and attitudes did not meet the acceptable AVE threshold but their composite reliability scores (above 0.60) indicate that convergent validity was still adequate [36]. Additionally, we conducted variance inflation factor (VIF) and tolerance tests to determine whether there was evidence of multicollinearity in the factors with lower AVE scores. Those tests yielded acceptable VIF scores ranging from 1.06–2.18 and acceptable tolerance scores ranging from 0.46 to 0.94, therefore we accepted that there was sufficient evidence of convergent validity without multicollinearity. Results from the tests for construct reliability and convergent validity are reported in Appendix B.

### 2.2. Survey Sample

The survey was conducted with a random sample of Alberta businesses from InfoCanada’s database of Canadian enterprises. The survey was designed to be completed by a staff member in charge of or authorized to make decisions about their organization’s workplace health (henceforth known as employers); this includes owners, general managers, human resource managers, and occupational health and safety managers. To ensure sufficient sample size to conduct stratified analyses, large organizations (defined as 250 or more employees in our sampling frame) were oversampled with the goal of representing 10% of the sample (see Appendix C for details on sampling frame). The margin of error for the difference between businesses sampled differed and the general population was ±4.37%.

### 2.3. Ethics

Ethics approval was obtained from the Health Research Ethics Board at the University of Alberta. All data has been securely collected, maintained, stored, transmitted, and reported in accordance with the Freedom of Information and Protection of Privacy Act (FOIP) and Personal Information Protection Act (PIPA).

### 2.4. Data Collection

The survey was administered between November 2017 and January 2018 by Computer-Assisted Telephone/Web/Personal Interview (CATI/CAWI/CAPI) systems, advanced optical mark recognition (OMR) and analysis software. The survey used mixed-mode delivery. Respondents could complete the survey over the phone, with trained surveyors, or online. Employers were first contacted by email to complete the survey online by 7 November 2017. Telephone surveying began on 7 November 2017and ended on 12 January 2018. In total, 2500 businesses were contacted to complete the survey, 472 of those businesses were considered invalid due to a variety of reasons including but not limited to: that the business no longer existed, the number was not in service, the number was incorrect, and the number was a fax or modem line. Of the 2028 valid calls, 528 businesses agreed to complete the survey yielding a response rate of 26.04%.

### 2.5. Data Analysis

Univariate statistics (percent agreement and sample size) are presented by survey question and organization size. Due to small sample size at the extremes of the scales, survey questions with degree of agreement responses were dichotomized (e.g., “strongly agree” and “agree” were coded as agree, and “strongly disagree” and “disagree” were coded as disagree). For frequency questions, “often” and “sometimes” were collapsed into one group and “rarely” and “never” were collapsed into another.

Latent Class Analysis (LCA) is a finite mixture model that uses an iterative approach on a set of categorical items to identify latent subgroups based on response patterns. Respondents are then assigned maximum posterior probabilities of membership in one of the exhaustive and mutually exclusive latent classes [37]. This technique allows for identification of respondent groups with similar knowledge, attitudes, practices, and likelihood of action for WHP. Four fields of interest were identified using LCA: (1) knowledge of WHP (8 items), (2) attitudes towards implementation of WHP (13 items), (3) practices for implementation of WHP (7 items), and (4) likelihood of action of WHP in the organization (8 items) (for more detail on the items included see Appendix A). Responses to these items were used to group respondents into latent classes with respondents with similar response profiles. Akaike Information Criteria (AIC), Bayesian Information Criteria (BIC), entropy index, and the relative parsimony and interpretability of latent classes were compared to select the final LCA models for the 4 fields of interest.

Latent class membership for the four fields of interest was regressed on 3-level organization size using a multinomial logistic model to produce odds of membership. Odds ratios and corresponding p-values were estimated. Latent classes representing high knowledge, positive attitudes, high practices, and high likelihood of action were used as reference groups. A 3-level organization size (<20 employees, 20–99 employees, 100+ employees) was used as the explanatory variable for all regression analyses, where organizations with more than 100 employees was the reference group.

Analyses was performed using SAS [version 3.6] and R [version 1.1.463]. LCA and multinomial logistic regression was performed using the poLCA package in R [38].

## 3. Results

A total of 528 employers completed the survey. Table 1 presents the percentage of organizations surveyed by size and affirmative response to WHP knowledge, attitudes, practices, and likelihood of action questions, by organization size. Overall, employers had good knowledge of WHP (as measured through correct responses to the knowledge questions) and positive attitudes towards WHP (as measured through agreement with statements on the positive benefits of WHP). In comparison to knowledge and attitudes, practices for WHP and likelihood of WHP action were relatively low. Only 54% (49%–58%) of employers agreed that they had the funding and 61% (57%–66%) of employers agreed they had the human resources to plan and implement a WHP. Despite limited practices, 76% (72%–80%) of employers agreed they had management’s support to plan and implement a workplace health program. In practice, 64% (60%–68%) of employers offered a WHP. When examining percent agreement by organization size, we see a lower percent of agreement with the knowledge, attitudes, practices and likelihood of action questions among smaller organizations. Among the practice questions, smaller organizations were less likely to agree they had the funding to implement WHP 44% (38%–49%), the previous experience to plan and implement WHP 53% (48%–59%), and the previous experience to evaluate WHP 49% (44%–55%), and were less likely to agree they had WHP currently in place 53% (48%–59%), compared to medium and large organizations.

**Figure 2:**
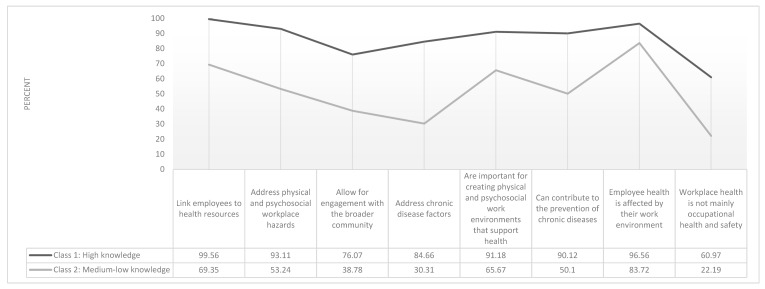
Percent Likelihood of Class Assignment Based on Affirmative Response to Eight Workplace Health Program (WHP) Knowledge Items (*n* = 528).

A two-class model was chosen to describe patterns of responses to eight WHP knowledge items. Figure 2 presents the item response probabilities (i.e., the probability that an individual belonging to a latent class will answer in the affirmative to each knowledge item) to eight knowledge items. Class 1 (70.37% of sample) is labelled “High Knowledge” as it includes respondents with high overall knowledge of WHP. Class 2 (29.27% of sample) is labelled “Medium–Low Knowledge” as it includes respondents with fewer correct responses to the WHP knowledge questions.

**Figure 3:**
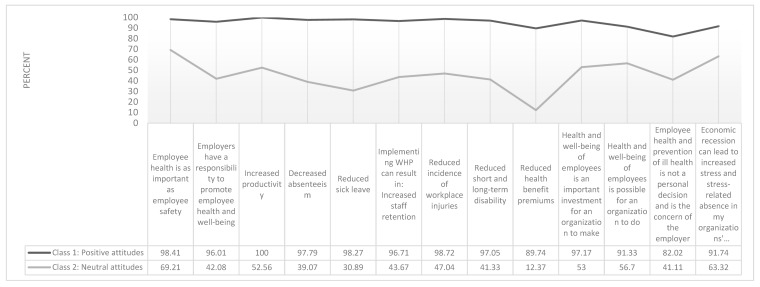
Percent Likelihood of Class Assignment Based on Affirmative Response to 13 WHP Attitude Items (*n* = 528).

A two-class model was also chosen to describe patterns of responses to 13 WHP attitude items. Figure 3 presents the item response probabilities (i.e., the probability that an individual belonging to a latent class will answer in the affirmative to each attitude item) to 13 attitudes variables. Class 1 (94.41% of sample) is labelled “Positive Attitudes” as it includes respondents with positive attitudes towards WHP. Class 2 (5.59% of sample) is labelled “Neutral Attitudes” as it includes respondents with fewer affirmative responses to the WHP attitude questions.

**Figure 4:**
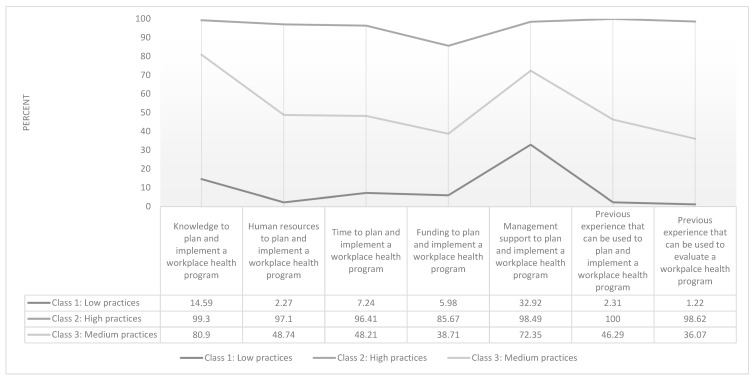
Percent Likelihood of Class Assignment Based on Affirmative Response to Seven WHP Practices Items (*n* = 528).

A three-class model was chosen to describe patterns of responses to seven WHP practices related items. Figure 4 presents the item response probabilities (i.e., the probability that an individual belonging to a latent class will answer in the affirmative to each practices item) to 8 practices variables. Class 1 (22.24% of sample) is labelled “Low Practices” as it includes respondents with far fewer affirmative responses to variables indicating practices in place to implement WHP. Class 2 (46.51% of sample) is labelled “High Practices” as it includes respondents with many affirmative responses to the variable indicating practices in place to implement WHP. Class 3 (32.25% of the sample) is labelled “Medium Practices” as it includes respondents with some affirmative responses to the variable indicating practices in place to implement WHP.

**Figure 5:**
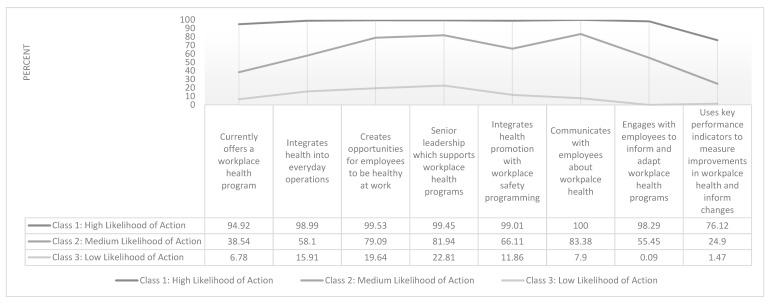
Percent Likelihood of Class Assignment Based on Affirmative Response to Eight WHP Likelihood of Action Items (*n* = 528).

Finally, a three-class model was chosen to describe patterns of responses to eight WHP likelihood of action items. Figure 5 presents the item response probabilities (i.e., the probability that an individual belonging to a latent class will answer in the affirmative to each likelihood of action item) to eight likelihood of action variables. Class 1 (53.72% of sample) is labelled “High Likelihood of Action” as it includes respondents with many characteristics associated with successful implementation of WHP in their organization. Class 2 (31.13% of sample) is labelled “Medium Likelihood of Action” as it includes respondents with some many characteristics associated with successful implementation of WHP in their organization. Class 3 (15.15% of the sample) is labelled “Low Likelihood of Action” as it includes respondents with few many characteristics associated with successful implementation of WHP in their organization.

Table 2 includes the odds ratios and corresponding *p*-values from a multinomial regression, estimating the effect of 3-level organization size on predicting membership in latent classes of the 4 fields of interest: (1) knowledge about WHP, (2) attitudes towards WHP, (3) practical capacity for WHP, and (4) WHP likelihood of action. Large organizations (100 or more employees) is used as the reference group for organization size and High Knowledge, Positive Attitudes, High Practical Capacity*,* and High Likelihood of Action are used as the reference groups for the latent classes of knowledge, attitudes, practices capacity, and likelihood of action, respectively. Compared to organizations with 100 or more employees, organizations with fewer than 20 employees were less likely (OR: 0.065; 95% CI: 0.0083–0.52) to be members of the Medium–Low Knowledge class compared to the High Knowledge class. There were no differences observed in the odds of class membership in the Positive Attitudes class, by organizations size. Compared to organizations with 100 or more employees, organizations with fewer than 20 employees were substantially more likely (OR: 12.37; 95% CI: 3.33–46.83) to be members of the Low Practices Capacity class compared to the High Practices class. Similarly, when compared to organizations with 100 or more employees, organizations with 20–99 employees were more likely to be members of the Low Practices class compared to the High Practices class. Finally, when compared to organizations with 100 or more employees, organizations with fewer than 20 employees were more likely to be members of the Medium Likelihood of Action class (OR: 2.36; 95% CI: 1.12–4.94) and the Low Likelihood of Action class (OR: 2.97; 95% CI: 1.10–8.00), compared to the High Likelihood of Action class.

## 4. Discussion

The present study surveyed 528 employers from organizations of varying size and industry across Alberta and measured their knowledge, attitudes, practices, and likelihood of action related to WHP. Approximately 64% of organizations currently offer WHP. Overall, Alberta organizations had strong knowledge of WHP and positive attitudes towards implementing WHP. Small organizations (<20 employees) were less likely, compared to large organizations, to be members of the Medium–Low Knowledge class compared to the High Knowledge class. This finding indicates that small organizations have good knowledge of WHP and what is included in a comprehensive WHP. Similarly, qualitative interviews with small employers in Western Australia recognized workplace health as a comprehensive program including occupational health and safety, mental health, nutrition, physical activity and general health [39].

Odds of membership in the Neutral Attitudes class compared to the Positive Attitudes class did not vary based on organization size. This result, combined with the finding that more than 94% of the sample were assigned to the Positive Attitudes class, indicates that organizations of any size view employee health as the employer’s responsibility and see the value of implementing WHP from the employee and employer perspective. A qualitative study carried out in Sweden found that managers of small organizations were aware of the benefits of workplace health programs and wished they could implement them but felt they needed additional support from organizations outside of the company [40]. Our results similarly indicated that employers of organizations with fewer than 20 employees had sufficient knowledge about the effectiveness and impact of WHP on the health of their employees but lacked the organizational practices to implement them. Compared to large organizations, small organizations had 12.37 times the odds of being members of the Low Practices class compared to the High Practices class. Medium organizations, compared to large organizations, had 4.76 times the odds of being members of the Low Practices class compared to the High Practices class. These findings support the results of a similar study conducted in Australia using a small business cut-off of fewer than 20 employees [16,29]. The recently published Centers for Disease Control (CDC) study found similar results as presented in this paper using a cut-off point for small business of under 50 employees (1,14). Our findings further highlight that organizations with fewer than 20 employees were less likely to report that they had sufficient practical capacity (funding, human resources, experience) to be able to implement a workplace health program compared to organizations with more employees. Interestingly, the CDC study found that the challenges of “cost” did not vary based on workplace size, which contradicts the findings of this study. Moreover, the CDC study found no difference in leadership commitment based on business size while the present study found small workplaces were more likely to be members of the Low Practices class (which includes items such as leadership commitment and tangible supports) compared to larger workplaces (1,14). These differences in findings may be due to the existence of incentivization schemes in the USA which do not exist in Canada. The Affordable Care Act in the USA created incentives to promote employer wellness programs with a maximum reward to employers of 30% of health coverage [41]. This incentivization model may have the dual benefit of reducing inequities in WHP funding between organizations of different sizes and increasing the level of leadership support for the implementation of WHP in practice.

Compared to large organizations, small organizations were 2.36 times more likely to be members of the Medium Likelihood of Action class and 2.97 times more likely to be members of the Low Likelihood of Action class compared to the High Likelihood of Action class. Small organizations experience substantial differences in terms of capacity for implementation and this is reflected in differences in WHP currently in place. These findings are supported by Biswas and associates [42] who found small workplaces (defined as less than 100 employees) had difficulties assigning resources and dedicated staff for wellness initiatives, perceived lack of employee interest and poor access to health promotion resources and wellness providers.

The present study has a number of strengths—namely, it is one of the few studies to examine knowledge, attitudes, practices, and likelihood of action for WHP, among small organizations, in Canada [32,41] and is the only study to do so using a context informed categorization of organization size which takes into account the demographic and legislative realities of the organizations in the region under study. Thus, these findings may be helpful for workplace health practitioners in other jurisdictions who face similar realities. The use of the under 100 employee cut-off in Canada, or other equally inappropriate cut-offs, may be masking inequities in knowledge, attitudes, practices, and likelihood of action for workplace health programs, among organizations that have less than 20 employees. Organizations with fewer than 20 employees make up the vast majority of organizations in Alberta, but they are typically not the focus of research. Finally, this study uses LCA, an exploratory dimensional reduction technique, which also accounts for higher order interactions between categorical items yet has a low probability of Type I error and provides higher statistical power [38]. In other words, using LCA reduces the possibility of Type I error due to repeated tests.

The limitations of the present study include the use of a cross-sectional study where causal relationships cannot be inferred and the use of self-reported measures which are susceptible to recall bias and social desirability bias. The decision to dichotomize 4-level Likert scales is a limitation of the study as it results in reduction of variance in responses but was necessary due to low response in the extremes of the scale. To the authors’ knowledge, no standardized tools for assessing knowledge, attitudes, practices and likelihood of action for workplace health implementation exists. However, our survey instrument is informed by an established theoretical framework [26,27] and years of research on workplace health promotion [3,28,29,30,31,32,33]. In addition, we assessed the construct reliability and convergent validity using Cronbach’s alpha and factor loadings, respectively. Another limitation in the present study focuses on organizational size as the exposure variable but does not include industry type. The examination of potential interactions between size and industry and the impact on knowledge, attitudes, practices, and likelihood of action for workplace health is an area for future research but is beyond the scope of the present study.

The results of this study present areas for further research in the context of implementing workplace health programs among small organizations in Canada, and other jurisdictions with similar demographic characteristics. While employers agreed (97%) that workplace health is as important as workplace safety, this attitude did not translate into high disagreement (50%) with the statement, “workplace health is mainly occupational health and safety, ensuring that workers are safe from hazards on the job”. Workplaces may not be making the connection between workplace conditions and health as readily as they are making the connection between employee safety and injuries. Alternatively, safety-focused industries might be more likely to agree with the above statement compared with non-safety focused industries. Recent research on the co-occurrence of occupational health and safety activities and health promotion activities found that 84% of workplaces in Ontario had little or no co-occurrence and that higher co-occurrence was related to workplace size, industry and workplace values [42]. Further research should examine the interaction between organization size and industry on knowledge, attitudes, practices, and likelihood of action for workplace health programs. It is clear from the results of this study that small organizations understand the value of workplace health programs but lack the organizational practices to implement them in reality. Results from in-depth interviews with managers of small organizations highlight the need for additional external supports outside of the company [40]. Additionally, other researchers have emphasized the need for government policy interventions and incentives for small workplaces to increase the implementation of WHP [6,43,44] and results from the recent CDC study may indicate the success of such incentivization models for decreasing the funding differences between organization sizes [1,14]. Therefore, there is a need for a systems-level analysis of incentivization approaches and to examine the feasibility and transferability of these structural approaches to a Canadian, or provincial context.

## 5. Conclusions

The vast majority of workplace health promotion literature focused on small workplaces is based in the USA which has limited applicability to Canada, given organizations in the USA are larger in size and use a different health care system (i.e., employer paid health benefits) [12]. The present study used three cut-offs to defined organization size (<20 employees, 20–99 employees, 100 or more employees) this was done to capture the unique demographic and legislative context of Alberta organizations. Few research studies conducted on small organizations in Canada define small organizations with precise cut-offs that reflect the demographic and legislative contexts of the location in which the research is being conducted. The use of an imprecise small business cut-off is masking the inequities in knowledge, attitudes, practices, and likelihood of action for WHP found in organizations with fewer than 20 employees, by collapsing them into a group that do not experience the same barriers. Overall, practical barriers such as funding prevent smaller workplaces from implementing WHP despite knowledge and attitudes in support of implementation. Evidence from the USA would suggest that incentivization of WHP could decrease some of the differences in funding by organization size [1,14]. Further analysis is needed to examine the systems level supports and barriers that can be developed to support these organizations in implementing WHP.

## Figures and Tables

**Table 1:** Percent Distribution and 95% Confidence Interval of Alberta Employer Affirmative Response to WHP Knowledge, Attitudes, Practices, and Likelihood of Action Questions by Organizational Size (*n* = 528).

Organization Size (*n* = 528)	Any Size	<20 Employees	20–99 Employees	100+ Employees
**Knowledge (*n* = 528) ** **Percent Responding “True”**				
Link employees to health resources	91% (88%–93%)	86% (82%–90%)	96% (93%–99%)	99% (96%–100%)
Address physical and psychosocial (such as stress, bullying and violence) workplace hazards	81% (78%–85%)	77% (72%–81%)	85% (80%–91%)	93% (87%–99%)
Allow for engagement with the broader community/assisting surrounding communities with their development initiatives or projects	65% (61%–69%)	63% (58%–69%)	63% (56%–71%)	77% (67%–87%)
Address chronic disease factors such as healthy eating, physical activity levels and tobacco cessation	69% (65%–73%)	64% (59%–70%)	69% (62%–76%)	87% (79%–95%)
Are important for creating physical and psychosocial work environments that support health	84% (75%–82%)	80% (76%–85%)	88% (83%–93%)	89% (81%–96%)
Can contribute to the prevention of chronic diseases such as diabetes, cancer, high blood pressure and arthritis	78% (81%–87%)	72% (67%–78%)	86% (81%–91%)	86% (77%–94%)
Employee health is affected by their work environment	93% (91%–95%)	92% (88%–95%)	94% (90%–98%)	96% (91%–100%)
Disagreement with: Workplace health is mainly occupational health and safety, ensuring that workers are safe from hazards on the job	50% (45%–54%)	46% (40%–51%)	51% (43%–58%)	64% (53%–76%)
**Attitudes (*n* = 528) ** **Percent Responding “Strongly Agree” or “Agree”**				
Employee health is as important as employee safety	97% (95%–98%)	96% (94%–98%)	97% (94%–100%)	99% (96%–100%)
Employers have a responsibility to promote employee health and well-being	93% (91%–95%)	92% (89%–95%)	93% (89%–97%)	97% (93%–100%)
Implementing activities to support the health and well-being of employees can result in: Increased productivity	97% (96%–99%)	96% (94%–98%)	99% (98%–100%)	99% (96%–100%)
Decreased absenteeism	95% (93%–96%)	94% (91%–96%)	96% (93%–99%)	94% (89%–100%)
Reduced sick leave	95% (93%–96%)	94% (91%–97%)	95% (92%–98%)	96% (91%–100%)
Increased staff retention	94% (92%–96%)	93% (89%–96%)	95% (92%–98%)	96% (91%–100%)
Reduced incidence of workplace injuries	96% (94%–98%)	95% (93%–98%)	96% (93%–99%)	97% (93%–100%)
Reduced short and long-term disability	94% (92%–96%)	93% (90%–96%)	95% (91%–98%)	97% (93%–100%)
Reduced health benefit premiums	85% (82%–88%)	83% (79%–88%)	87% (82%–92%)	90% (83%–97%)
Implementing activities to support the health and well-being of employees is an important investment for an organization to make	95% (93%–97%)	94% (91%–97%)	95% (92%–98%)	97% (93%–100%)
Implementing activities to support the health and well-being of employees is possible for an organization to do	89% (87%–92%)	87% (83%–91%)	90% (86%–95%)	97% (93%–100%)
Disagreement with: Employee health and prevention of ill health are a personal decision and is not the concern of the employer	80% (76%–83%)	78% (73%–83%)	80% (74%–86%)	87% (79%–95%)
Economic recession can lead to increased stress and stress-related absence in my organizations’ employees	90% (88%–93%)	88% (85%–92%)	91% (87%–96%)	94% (89%–100%)
**Practices (*n* = 528)** **Percent Responding “Strongly Agree” or “Agree”**				
The knowledge to plan and implement a workplace health program	75% (72%–79%)	68% (63%–74%)	80% (74%–86%)	94% (89%–100%)
The human resources (dedicated staff) to plan and implement a workplace health program	61% (57%–66%)	53% (48%–59%)	65% (58%–73%)	86% (77%–94%)
The time to plan and implement a workplace health program	62% (58%–66%)	56% (51%–62%)	65% (58%–73%)	77% (67%–87%)
The funding to plan and implement a workplace health program	54% (49%–58%)	44% (38%–49%)	65% (58%–73%)	69% (57%–80%)
The management support to plan and implement a workplace health program	76% (72%–80%)	73% (68%–78%)	81% (74%–87%)	79% (69%–88%)
Previous experience that can be used to plan and implement a workplace health program	62% (58%–66%)	53% (48%–59%)	70% (62%–77%)	80% (70%–90%)
Previous experience that can be used to evaluate a workplace health program	58% (54%–62%)	49% (44%–55%)	64% (57%–71%)	79% (69%–88%)
**Likelihood of Action (*n* = 528)** **Percent Responding “Sometimes” or “Often”**				
Currently offers a workplace health program	64% (60%–68%)	53% (48%–59%)	75% (68%–82%)	83% (74%–92%)
Integrates health into everyday operations	74% (70%–77%)	71% (66%–76%)	76% (70%–83%)	79% (69%–88%)
Creates opportunities for employees to be healthy at work	81% (78%–84%)	79% (74%–83%)	84% (78%–89%)	86% (77%–94%)
Senior leadership which supports workplace health programs	82% (79%–86%)	79% (74%–84%)	84% (78%–89%)	94% (89%–100%)
Integrates health promotion with workplace safety programming	76% (72%–79%)	71% (66%–76%)	80% (74%–86%)	84% (76%–93%)
Communicates with employees about workplace health	81% (78%–84%)	78% (73%–83%)	85% (80%–91%)	83% (74%–92%)
Engages with employees to inform and adapt workplace health programs	70% (66%–74%)	66% (61%–72%)	73% (66%–80%)	79% (69%–88%)
Uses key performance indicators to measure improvements in workplace health and inform changes	49% (45%–53%)	44% (38%–50%)	54% (47%–62%)	57% (45%–69%)

**Table 2:** Estimated Odds Ratio from a Multinomial Logistic Regression of Membership in Latent Classes of WHP Knowledge, Attitudes, Practices, and Likelihood of Action on Organization Size (*n* = 528).

	Model 1: Knowledge (ref: High Knowledge)	Model 2: Attitudes (ref: Positive Attitude)	Model 3: Practices (ref: High Practices)		Model 4: Likelihood of Action (ref: High Likelihood of Action)	
	Medium–Low Knowledge	Neutral Attitude	Low Practices Capacity	Medium Practices Capacity	Medium Likelihood of Action	Low Likelihood of Action
<20 employees	0.065 * (0.0083–0.52)	1.20 (0.099–11.51)	12.37 ** (3.33–46.83)	1.81 (0.92–3.43)	2.36 * (1.12–4.94)	2.97 * (1.10–8.00)
20 to 99 employees	0.18 (0.022–1.45)	2.68 (0.30–17.17)	4.76 * (1.21–19.31)	1.41 (0.68–2.76)	1.21 (0.56–2.61)	1.56 (0.53–4.60)
≥100 employees (ref)	-	-	-	-	-	-

* *p* < 0.05; ** *p* < 0.001.

## Appendix A

**Table A1:** Workplace Health KAP Survey Instrument Including Question Scale and KAP Concepts from Conceptual Model.

**Knowledge**	**Scale**	**KAP concept**
Workplace health programs link employees to health resources	True/False	Awareness-impact
Workplace health programs do not address physical and psychosocial (such as stress, bullying or violence) workplace hazards	True/False	Awareness-knowledge
Workplace health programs allow for engagement with the broader community/assisting surrounding communities with their development initiatives or projects	True/False	Awareness-knowledge
Workplace health programs address chronic disease factors such as healthy eating, physical activity levels and tobacco cessation	True/False	Awareness-impact
Workplace health programs are not important for creating physical and psychosocial work environments that support health	True/False	Awareness-impact
Workplace health programs can contribute to the prevention of chronic diseases such as diabetes, cancer, high blood pressure and arthritis	True/False	Awareness-impact
Employee health is not affected by their work environment	True/False	Awareness-impact
Workplace health is mainly occupational health and safety, ensuring that workers are safe from hazards on the job	True/False	Awareness-knowledge
**Attitudes**	**Scale**	**KAP concept**
Employee health is as important as employee safety	Likert: strongly agree, agree, disagree, strongly disagree	Agreement-justification
Employers have a responsibility to promote employee health and wellbeing	Likert: strongly agree, agree, disagree, strongly disagree	Motivation-responsibility
Implementing activities to support the health and wellbeing of employees can result in: Increased productivity Decreased absenteeism Increased staff retention Reduced incidence of workplace injuries Reduced short and long-term disability costs Reduced health benefit premiums	Likert: strongly agree, agree, disagree, strongly disagree	Outcome expectation
Implementing activities to support the health and wellbeing of employees is an important investment for an organization to make	Likert: strongly agree, agree, disagree, strongly disagree	Agreement, motivation
Implementing activities to support the health and wellbeing of employees is possible for an organization to do	Likert: strongly agree, agree, disagree, strongly disagree	Perceived self-efficacy
Employee health and prevention of ill health are a personal decision and is not the concern of the employer	Likert: strongly agree, agree, disagree, strongly disagree	Agreement, motivation
Economic recession can lead to increased stress and stress related–absence in my organization’s employees	Likert: strongly agree, agree, disagree, strongly disagree	Contextual factor
**Practices**	**Scale**	**KAP concept**
My organization has the knowledge to plan and implement a workplace health program	Likert: strongly agree, agree, disagree, strongly disagree	Perceived self-efficacy
My organization as the human resources (dedicated staff) to plan and implement a workplace health program	Likert: strongly agree, agree, disagree, strongly disagree	Perceived self-efficacy
My organization has the time to plan and implement a workplace health program	Likert: strongly agree, agree, disagree, strongly disagree	Feasibility
My organization has the funding to plan and implement a workplace health program	Likert: strongly agree, agree, disagree, strongly disagree	Perceived self-efficacy
My organization has the management support to plan and implement a workplace health program	Likert: strongly agree, agree, disagree, strongly disagree	Feasibility
My organization has previous experience that can be used to plan and implement a workplace health program	Likert: strongly agree, agree, disagree, strongly disagree	Perceived self-efficacy
My organization has previous experience that can be used to evaluate a workplace health program	Likert: strongly agree, agree, disagree, strongly disagree	Perceived self-efficacy
**Likelihood of Action**	**Scale**	**KAP concept**
My organization currently offers a workplace health program	Likert: often, sometimes, rarely, never	Action
My organization integrated health into everyday operations	Likert: often, sometimes, rarely, never	Feasibility
My organization creates opportunities for employees to be healthy at work	Likert: often, sometimes, rarely, never	Feasibility
My organization’s senior leadership supports workplace health programs	Likert: often, sometimes, rarely, never	Feasibility
My organization integrates health promotion with workplace safety programming	Likert: often, sometimes, rarely, never	Feasibility
My organization communicates with employees about workplace health	Likert: often, sometimes, rarely, never	Feasibility
My organization engages with employees to inform and adapt workplace health programs	Likert: often, sometimes, rarely, never	Feasibility
My organization uses key performance indicators to measure improvements in workplace health and inform changes	Likert: often, sometimes, rarely, never	Feasibility

## Appendix B

**Table A2:** Cronbach’s Alpha, Factor Loading and Convergent Validity.

Knowledge			Attitudes			Practices		Likelihood of Action	
Items Included	Factor Loadings		Items Included	Factor Loadings		Items Included	Factor Loadings	Items Included	Factor Loadings
	Factor 1	Factor 2		Factor 1	Factor 2		Factor 1		Factor 1
Item 1	0.58	-	Item 1	0.52	-	Item 1	0.76	Item 1	0.72
Item 2	-	0.53	Item 2	-	0.48	Item 2	0.79	Item 2	0.74
Item 3	0.63	-	Item 3	-	-	Item 3	0.76	Item 3	0.73
Item 4	0.75	-	Item 4	0.78	-	Item 4	0.69	Item 4	0.75
Item 5	-	0.72	Item 5	0.74	-	Item 5	0.67	Item 5	0.76
Item 6	0.51	-	Item 6	0.77	-	Item 6	0.84	Item 6	0.81
Item 7	-	0.75	Item 7	0.57	-	Item 7	0.83	Item 7	0.81
Item 8	0.49	-	Item 8	0.72	-	Item 8	-	Item 8	0.63
			Item 9	0.75	-				
			Item 10	0.57	-				
			Item 11	-	0.68				
			Item 12	-	0.65				
			Item 13	-	0.69				
			Item 14	-	0.42				
AVE	0.36	0.45		0.47	0.35		0.59		0.56
Composite Reliability	0.73	0.71		0.87	0.73		0.91		0.91

## Appendix C

**Table A3:** Actual and Projected Sample Frame by Organization Size and Industry Type (*n* = 528).

Variable	Sample Percentage	Sample Frame
Organization Size		
Fewer than 20 employees	56%	60%
20 to 249 employees	34%	30%
250 or more employees	10%	10%
Industry (NAICS code)		
Agriculture (11)	2.1%	3.4%
Mining and oil and gas extraction (21)	1.7%	3.4%
Utilities (22)	3.0%	3.4%
Construction (23)	8.1%	8.0%
Manufacturing (31–33)	5.9%	5.6%
Wholesale trade (41)	8.5%	8.0%
Retail trade (44–45)	8.7%	8.0%
Transportation and warehousing (48–49)	5.1%	3.4%
Information and culture industries (51)	3.8%	3.4%
Finance and insurance (52)	2.8%	3.6%
Real estate (53)	3.0%	3.4%
Professional, scientific and technical services (54)	8.0%	8.0%
Management of companies (55)	2.1%	3.4%
Administrative and support (56)	2.5%	3.5%
Educational services (61)	6.1%	3.4%
Health care and social assistance (62)	8.3%	6.9%
Arts, entertainment and recreation (71)	3.0%	3.4%
Accommodation and food services (72)	7.6%	6.6%
Other services (excl. public administration) (81)	6.6%	8.0%
Public administration (91)	3.0%	3.4%
